# Characterization of the *C. elegans *erlin homologue

**DOI:** 10.1186/1471-2121-13-2

**Published:** 2012-01-23

**Authors:** Maja B Hoegg, Stephen M Robbins, James D McGhee

**Affiliations:** 1 Department of Biochemistry & Molecular Biology, University of Calgary, 3330 Hospital Drive NW, Calgary, Alberta T2N 4N1, Canada; 2 Department of Oncology, University of Calgary, 3330 Hospital Drive NW, Calgary, Alberta T2N 4N1, Canada; 3 Southern Alberta Cancer Research Institute, University of Calgary, 3330 Hospital Drive NW, Calgary, Alberta T2N 4N1, Canada; 4 Department of Medical Genetics, University of Calgary, 3330 Hospital Drive NW, Calgary, Alberta T2N 4N1, Canada; 5 Alberta Children's Hospital Research Institute, University of Calgary, 3330 Hospital Drive NW, Calgary, Alberta T2N 4N1, Canada

## Abstract

**Background:**

Erlins are highly conserved proteins associated with lipid rafts within the endoplasmic reticulum (ER). Biochemical studies in mammalian cell lines have shown that erlins are required for ER associated protein degradation (ERAD) of activated inositol-1,4,5-trisphosphate receptors (IP3Rs), implying that erlin proteins might negatively regulate IP3R signalling. In humans, loss of erlin function appears to cause progressive intellectual disability, motor dysfunction and joint contractures. However, it is unknown if defects in IP3R ERAD are the underlying cause of this disease phenotype, whether ERAD of activated IP3Rs is the only function of erlin proteins, and what role ERAD plays in regulating IP3R-dependent processes in the context of an intact animal or embryo. In this study, we characterize the erlin homologue of the nematode *Caenorhabditis elegans *and examine erlin function *in vivo*. We specifically set out to test whether *C. elegans *erlin modulates IP3R-dependent processes, such as egg laying, embryonic development and defecation rates. We also explore the possibility that erlin might play a more general role in the ERAD pathway of *C. elegans*.

**Results:**

We first show that the *C. elegans *erlin homologue, ERL-1, is highly similar to mammalian erlins with respect to amino acid sequence, domain structure, biochemical properties and subcellular location. ERL-1 is present throughout the *C. elegans *embryo; in adult worms, ERL-1 appears restricted to the germline. The expression pattern of ERL-1 thus only partially overlaps with that of ITR-1, eliminating the possibility of ERL-1 being a ubiquitous and necessary regulator of ITR-1. We show that loss of ERL-1 does not affect overall phenotype, or alter brood size, embryonic development or defecation cycle length in either wild type or sensitized *itr-1 *mutant animals. Moreover we show that ERL-1 deficient worms respond normally to ER stress conditions, suggesting that ERL-1 is not an essential component of the general ERAD pathway.

**Conclusions:**

Although loss of erlin function apparently causes a strong phenotype in humans, no such effect is seen in *C. elegans*. *C. elegans *erlin does not appear to be a ubiquitous major modulator of IP3 receptor activity nor does erlin appear to play a major role in ERAD.

## Background

Endoplasmic reticulum (ER) lipid raft associated proteins (erlins) were originally discovered by screening with antibodies prepared against isolated lipid raft proteins from human myelomonocytic cells [1]. Erlins associate with detergent resistant membranes but are located in the ER membrane, suggesting they are components of lipid raft-like domains in the ER membrane, not the plasma membrane. Erlins belong to the group of stomatin/prohibitin/flotillin/HflK/C (SPFH) domain containing proteins [1]. Members of this protein group differ in subcellular location and function, but share certain biochemical properties such as detergent resistant membrane association and the propensity to form oligomers [2].

Erlins are conserved in both plants and animals [3] but so far erlin proteins have only been studied experimentally in mammalian cell lines [1,3-5]. Interestingly, no erlin homologues are found in yeast or in *Drosophila melanogaster*. While *C. elegans *and *A. thaliana *have only one erlin gene, vertebrate species have two closely related erlin homologues [1,6]. For instance, human erlin-1 and erlin-2 (also known as SPFH1/KE04p and SPFH2/C8orf2 respectively) share ~80% identity at the amino acid level [1]. Erlins form large (1-2 MDa) higher order multimers, which is absolutely dependent on a single phenylalanine residue (F305 in human erlin-1 and -2) close to the C-terminus [4,5].

Biochemical studies in mammalian cell lines have revealed an important role for erlin proteins in targeting activated IP3Rs for ER-associated protein degradation (ERAD) [3,5,7]. ERAD mediates the degradation of ER proteins by the cytosolic ubiquitin proteasome system [8]. The main function of ERAD is the removal of misfolded proteins from the ER [8], which is particularly important under conditions of ER stress when protein folding is impaired [9]. Another function of ERAD is to control levels and thus the activity of specific substrate proteins, including IP3 receptors [10]. IP3 receptors are calcium release channels in the ER membrane, which become activated and open in response to IP3 binding [11]. Upon sustained stimulation by certain ligands, activated IP3 receptors are targeted for ERAD, which is thought to provide a mechanism of desensitizing cells to IP3 [12].

Upon their activation, IP3Rs become rapidly associated with erlin proteins [3,5]. Blocking erlin expression by RNA interference prevents degradation of activated IP3 receptors and increases IP3R levels under resting conditions. Overexpression of wild type erlin proteins enhances IP3R turnover. In addition, erlin mutants defective in high MW complex formation disrupt erlin complexes and have a dominant-negative effect on IP3R ERAD [5]. This latter finding also shows that formation of multimeric complexes is required for erlin function. In addition, erlin proteins seem to play a rather minor role in ERAD of certain other model substrates [3,7].

A frameshift mutation in the *erlin-2 *gene appears to cause a rare human autosomal recessive disorder characterized by progressive intellectual disability, motor dysfunction, joint contractures and vacuolization of leukocytes [13]. The frameshift mutation results in a truncated, likely dominant negative version of erlin-2 that is defective in high MW complex formation [4,5,13]. It remains to be determined whether defects in IP3R ERAD are the underlying cause of this disease phenotype. It is also possible that erlins could have some entirely unsuspected function.

We have turned to the nematode *C. elegans *to study erlin function in the context of an intact organism. *C. elegans *is an excellent model organism in which to study IP3 receptor signaling and ERAD. The *C. elegans *IP3 receptor ITR-1, which is highly similar to mammalian IP3 receptors, is expressed in a wide range of tissues [14], where it regulates a number of rhythmic behaviours, such as defecation and ovulation [15,16]. ITR-1 is also important during early embryonic development, where it controls migration of epidermal cells [17]. Changes in ITR-1 activity lead to altered defecation cycle length, reduced brood size and increased embryonic arrest [15-17]. Many components of the ERAD pathway are also conserved between *C. elegans *and mammals [18-23]. Mutations in proteins involved in ERAD can be easily detected in *C. elegans *as they increase ER stress levels and increase sensitivity to agents that induce ER stress [19,20,22-24].

The present study represents the first characterization of the *C. elegans *erlin protein ERL-1. We examine general properties of ERL-1, such as biochemistry, subcellular location and expression pattern. A *C. elegans *strain carrying a chromosomal deletion in the *erl-1 *gene is used to examine the effect of erlin deficiency on overall phenotype, specific IP3 receptor dependent processes and response to ER stress. Overall, our findings provide no evidence that C. elegans erlins play a major role either in modulating IP3R activity or in ERAD.

## Results

### The C. elegans protein ERL-1 is highly similar to human erlin-1 and erlin-2

**Figure 1:**
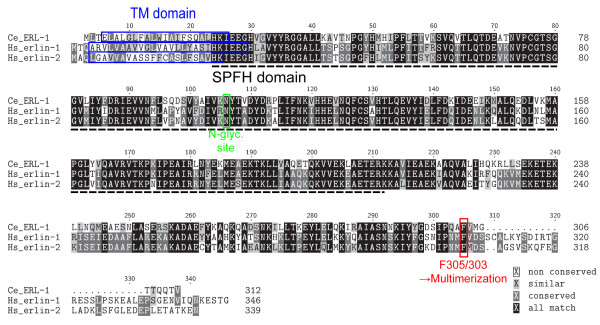
***C. elegans *ERL-1 is a homologue of human erlin proteins**. ClustalW alignment of *C.elegans *(Ce) ERL-1 and human (Hs) erlin-1 and erlin-2. Transmembrane domains (predicted by TMAP) are marked by blue boxes, N-glycosylation site is marked by green box, F305/303 required for oligomerization is marked by red box. The SPFH domain (pfam01145) is indicated by black dotted lines.

The *C. elegans *gene C42C1.15 (hereafter referred to as *erl-1*) encodes a 312 amino acid protein (ERL-1) with strong similarity to mammalian erlin-1 and erlin-2 (reciprocal blastp probabilities in the range of 10^-111 ^to 10^-115 ^) [3]. No other credible erlin homologue can be found in the *C. elegans *genome (next best reciprocal blastp probability ~ 0.001). Alignment of the amino acid sequences between *C. elegans *ERL-1 and either human erlin-1 or erlin-2 shows ~65% identity (73% similarity) (Figure 1). Several important features previously identified in mammalian erlins [1,3] are also present in the *C. elegans *protein, including the N-terminal transmembrane domain, the SPFH domain and the N-glycosylation site. In addition, a phenylalanine residue required for high molecular weight (MW) complex formation of human erlins (F305) [4], is conserved in *C. elegans *ERL-1 (F303).

**Figure 2:**
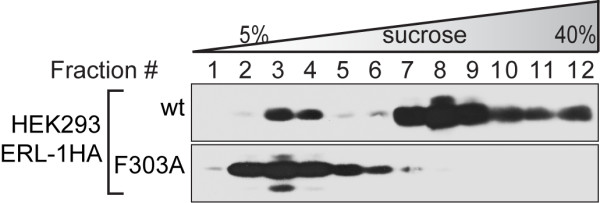
**ERL-1 association into high MW complexes depends on Phe-303**. Sucrose density gradient centrifugation was performed on HEK293 cells transiently transfected with wild type (upper panel) or F303A (lower panel) ERL-1HA. Twelve fractions were collected from each gradient, which were analyzed by Western blotting using an HA-tag specific antibody.

To confirm that ERL-1 forms complexes similar to its human counterparts, we performed sucrose gradient centrifugation on extracts of HEK293 cells transiently transfected with an HA-tagged version of *erl-1 *cDNA. As previously found for human erlin proteins [4], wild type ERL-1HA became enriched in higher MW fractions with a peak concentration in fraction 8 (Figure 2). Substitution of F303 with alanine (F303A) shifted ERL-1 into lower MW fractions, demonstrating that this residue is necessary for high MW complex formation of ERL-1 (Figure 2).

**Figure 3:**
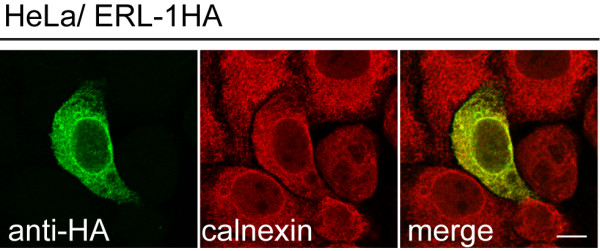
**Ectopically expressed ERL-1 localizes to the ER**. Confocal image of HeLa cell transiently transfected with ERL-1HA cDNA. Cells were stained with rat α-HA (green) and rabbit α-calnexin (red) antibodies. Scale bar = 10 μm.

To determine if *C. elegans *ERL-1 localizes to the ER, as do mammalian erlin proteins, we performed immunofluorescent staining of HA-tagged ERL-1 ectopically expressed in HeLa cells. Antibody staining of ERL-1HA revealed a cytoplasmic and perinuclear pattern that co-localized with the ER chaperone calnexin (Figure 3), indicating that *C. elegans *ERL-1 localizes to the ER and occupies the same subcellular compartments as mammalian erlin proteins.

Overall, *C. elegans *and human erlin proteins appear to be highly similar with respect to amino acid sequence, biochemical properties and subcellular location. It is thus reasonable to expect that erlin protein function is also conserved between the two species.

### ERL-1 is widespread in the embryo, but is primarily expressed in the gonad in adult worms

To determine the expression pattern of *erl-1*, we first attempted to use transcriptional GFP reporter constructs but this approach is complicated by the fact that *erl-1 *is part of an operon. However, a fraction of *erl-1 *transcripts contain SL1 trans-splice leaders, suggesting the possibility of operon-independent transcription [25,26]. Three different GFP reporter constructs were generated by cloning upstream regions of *erl-1 *(relative to the *erl-1 *start codon: -182 to +1; -1022 to +1; -1022 to +576) 5' of a GFP transgene (Additional File 1, Figure S1). However, none of these potential *erl-1 *promotor regions induced detectable GFP expression in transgenic worms (data not shown).

**Figure 4:**
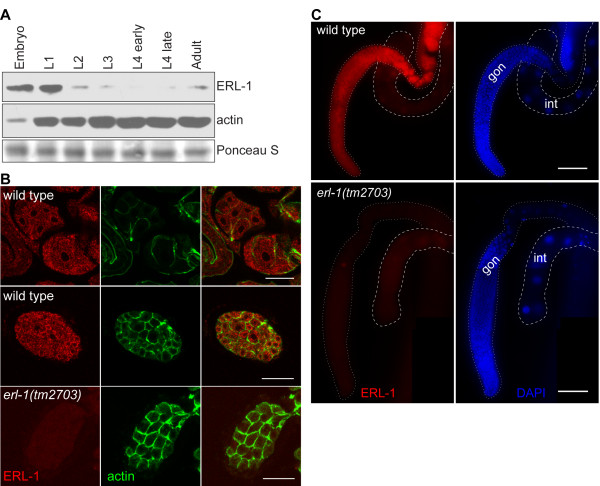
**ERL-1 expression during *C. elegans *development**. (A) ERL-1 protein expression levels at different stages of *C. elegans *development were examined by Western blotting. Equal amounts of protein were loaded for each developmental stage. Western blot probed for actin shows equal protein loading of larval and adult samples. Embryos express low levels of actin relative to total amount of protein; we therefore also show a Ponceau S stained band that has equal intensity at all developmental stages. (B) Confocal images of *C. elegans *embryos stained with rabbit α-ERL-1 (red) and mouse α-actin (green). Scale bar = 20 μm. (C) Fluorescent micrographs of dissected gonads (gon, outlined by white dotted lines) and intestines (int, outlined by white dashed lines) stained with rabbit α-ERL-1 (red) and DAPI (blue). Scale bar = 50 μm.

Antibodies were raised against the C-terminal half of the ERL-1 protein and used for Western blot analysis to identify developmental stages at which ERL-1 protein was expressed. ERL-1 could be detected throughout worm development, with highest expression levels (normalized to total protein) in embryos and L1 larvae (Figure 4A). Next we performed immunofluorescent staining of ERL-1 at various developmental stages. ERL-1 could be detected in the cytoplasm of all cells of early embryos (Figure 4B). In adult worms, ERL-1 staining was primarily observed in the gonad. Figure 4C shows staining of dissected adult gonads and intestines: Staining with ERL-1 antibody revealed that ERL-1 protein is expressed throughout the gonad but appears absent from the intestine, i.e. ERL-1 staining did not exceed the background levels observed with the *erl-1(tm2703) *null mutant animals described below.

Our finding that *erl-1 *is primarily expressed in the gonad of adult worms is consistent with previously published serial analyses of gene expression (SAGE) data. SAGE studies have detected *erl-1 *transcripts in dissected gonads [27] and purified oocytes [28] but neither in *glp-4(bn2) *animals (which lack gonads) nor in isolated *glp-4(bn2) *intestines [29]. Silencing of transgenes in the *C. elegans *germline [30] could explain why *erl-1 *gene expression could not be detected using transcriptional reporter constructs.

### ERL-1 deficiency does not cause an obvious phenotype

**Figure 5:**
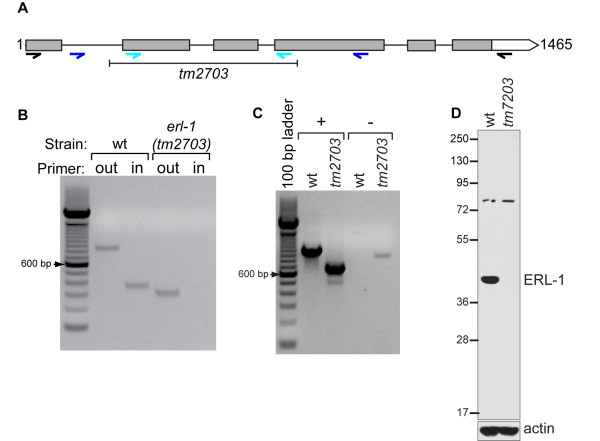
**Characterization of the *erl-1(tm2703) *allele**. (A) Schematic of *erl-1 *gene: grey and white boxes indicate exons and 3'UTR respectively. The *erl-1 *region deleted in *tm2703 *is marked by black line. Primers used for RT-PCR are shown as black arrows. Blue and cyan arrows indicate primers used to confirm *tm2703 *deletion. Primers binding outside and inside the deleted region are shown in blue and cyan respectively. (B) Genomic deletion in *erl-1(tm2703) *was confirmed by genomic PCR using primers that bind outside and inside the deleted region as shown in Figure 5A. (C) *erl-1 *mRNAs isolated from wild type and *erl-1(tm2703) *worms were amplified by RT-PCR. PCR was performed with either cDNA (+) or RNA (-) using primers depicted in Figure 4A. The weak band in *erl-1(tm2703) *RNA only sample (-) likely results from amplification of residual genomic DNA in the RNA preparation, i.e. the size of product corresponds to size of *erl-1 *genomic region. (D) Western blot analysis shows lack of ERL-1 protein in the strain homozygous for *erl-1(tm2703)*. ERL-1 was detected with affinity purified rabbit α-ERL-1 and blot was re-probed with mouse α-actin as loading control.

To explore the *in vivo *function of ERL-1 in *C. elegans*, we utilized a strain homozygous for the allele *tm2703*, a 536 bp deletion within the *erl-1 *gene that removes exons 2 and 3 and part of exon 4 (Figure 5A). The *tm2703 *allele is predicted to cause a frame shift leading to a premature stop codon. The resulting N-terminal 34 amino acid truncation product is unlikely to be functional, even if it were to be expressed.

We used genomic PCR with two different primer sets to confirm the presence of this deletion in a strain homozygous for *erl-1(tm2703) *(Figure 5B). RT-PCR and cDNA sequencing showed that the transcripts produced from wild type and mutant *erl-1 *genes contained the predicted sequences (Figure 5C). The absence of ERL-1 protein expression in *erl-1(tm2703) *mutants was demonstrated by Western blotting using an ERL-1 specific antibody. The ERL-1 antibody detected a band of ~40 kDa in wild type lysates that was completely absent in *erl-1(tm2703) *lysates, consistent with *tm2703 *being an *erl-1 null *allele

**Figure 6:**
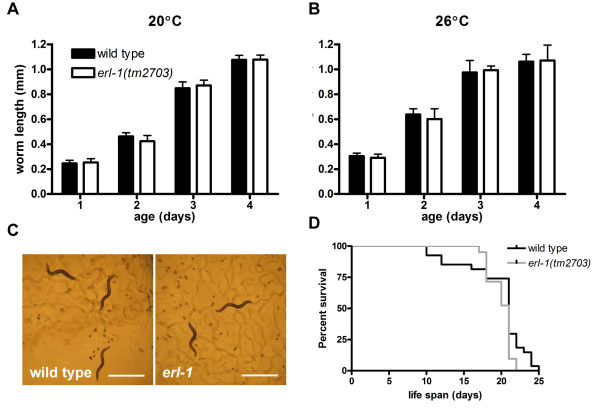
***erl-1(tm2703) *does not change overall phenotype of C. elegans**. (A and B) Body length of wild type and *erl-1(tm2703) *worms from one to four days of age. Worms were grown at either 20°C (A) or 26°C (B). Values represent the average length of 16 animals +/- SD. (B) Photographs of four day old worms, grown at 20°C. Scale bar = 1 mm. (C) Survival curve comparing life span of *erl-1(tm2703)*, n = 21, to life span of wild type, n = 27.

The role of ERL-1 in *C. elegans *has not been previously characterized. We therefore examined the overall phenotype of a strain homozygous for *erl-1(tm2703) *(following five rounds of outcrossing to remove extraneous mutations induced by the mutagenesis used to produce the deletion). There was no measureable difference in growth rate at 20°C (Figure 6A), in general morphology (Figure 6C), and in life span (Figure 6D) between *erl-1(tm2703) *and wild type worms. Even when worms were grown under heat stress conditions at 26°C, *erl-1(tm2703) *had no effect on growth rate (Figure 6B). Thus lack of ERL-1 has either no effect or a very minor effect on overall viability and phenotype of *C. elegans*.

### ERL-1 is not a major modulator of ITR-1 dependent processes

Mammalian erlins have been shown to be required for ERAD of activated IP3 receptors, and have therefore been proposed to negatively regulate IP3 signaling [3,5,7]. To test this proposal, we examined the effect of ERL-1 deficiency on three different IP3R-dependent processes in *C. elegans*.

#### (a) Brood Size

**Figure 7:**
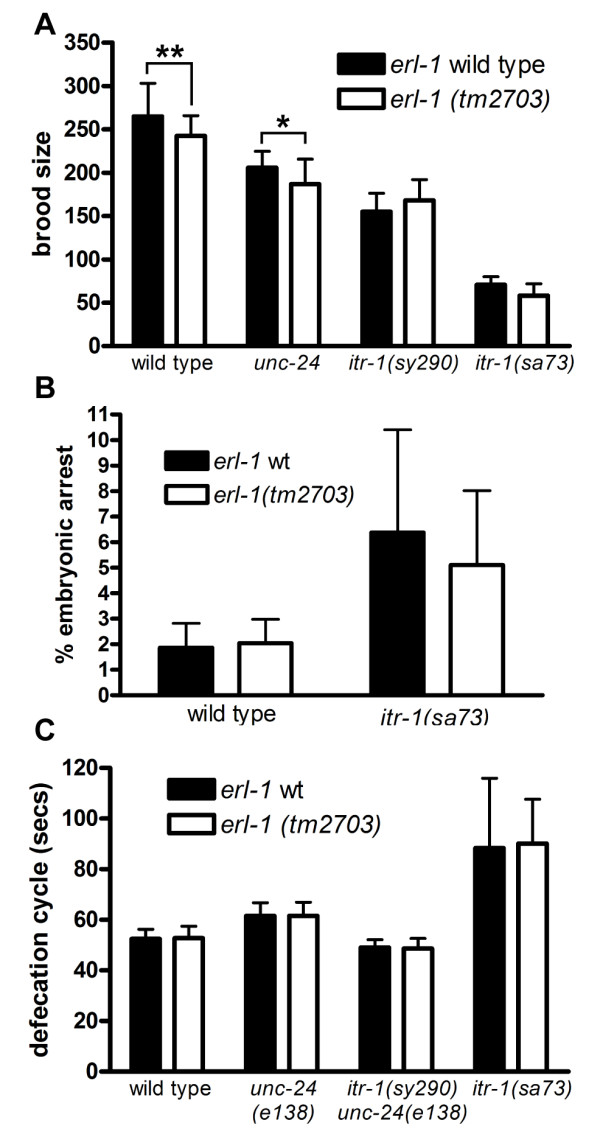
***erl-1(tm2703) *has no major effect on phenotype of *itr-1 *mutants**. The effect of *erl-1(tm2703) *on brood size (A), embryonic arrest (B) and defecation cycle length (C) on *wild type*, *unc-24(e138)*, *itr-1(sy290) unc-24(e138) *and/or *itr-1(sa73) *was measured. *sy290 *is a gain-of-function and *sa73 *is a weak loss-of-function allele of *itr-1*. Because *itr-1(sy290) *is closely linked to *unc-24(e138)*, the phenotype of *itr-1(sy290) unc-24(e138) *strains was compared to that of strains carrying *unc-24(e138) *alone. Black bars indicate *erl-1 *wild type and white bars indicate *erl-1(tm2703) *genotype. (A) Brood size was determined by counting the number of viable offspring per worm (n = 18-20; * indicates p-value < 0.05; ** indicates p-value < 0.01, one-way ANOVA and Newman-Keuls multiple comparison test). (B) Percentage of offspring arresting as embryos was determined. (C) Defecation cycle length was determined by measuring times between posterior body contractions (pBocs). Values represent the average (+/-SD) of six defecation cycles for each of ten worms (five worms for *itr-1(sa73) *strains).

Signaling through the *C. elegans *IP3 receptor ITR-1 is required for ovulation as it controls gonadal sheath cell contractions and spermathecal dilations [31,32]. Both increases and decreases in ITR-1 activity reduce brood size, albeit to a different extent [15,16]. For example, the weak *itr-1 *loss-of-function (LOF) allele *sa73 *reduces brood size by ~75%, while the *itr-1 *gain-of-function (GOF) allele *sy290 *reduces brood size by only ~25% (Figure 7A) [15,16]. We examined the effect of ERL-1 deficiency in both wild type and sensitized *itr-1 *mutant backgrounds. If ERL-1 were to be involved in ERAD of ITR-1, loss of ERL-1 should lead to increased ITR-1 levels and increased IP3R signaling: the overall effect would be that an *itr-1 *GOF phenotype should be enhanced and an *itr-1 *LOF phenotype should be suppressed. We found that *erl-1(tm2703) *slightly decreased brood size in a wild type background (*erl-1(tm2703)*: 242.5 ± 23; n = 18 vs. wild type: 265 ± 38; n = 19; p-value < 0.01) as well as in *unc-24(e138) *control worms (*unc-24(e138) erl-1(tm2703) *187 ± 29; n = 19 vs. *unc-24(e138) *206 ± 19; n = 20; p-value < 0.05) (Figure 7A). However, it would be difficult to ascribe this minor phenotype to the loss of erlin function, as opposed to a tightly linked but unrelated mutation not removed by outcrossing. More convincing is the finding that *erl-1(tm2703) *had no effect on brood size in *itr-1(sy290) unc-24 (e138) *double mutants or *itr-1(sa73) *mutants (Figure 7A). Thus, lack of ERL-1 has either no effect or has a very minor effect on the brood size in wild type animals and *itr-1 *mutants.

#### (b) Embryogenesis

ITR-1 also regulates epidermal cell migration, which is crucial during embryonic development. The weak *itr-1 *LOF allele *sa73 *increases rates of embryonic arrest by interfering with epidermal cell migration [17]. On average 2% of wild type embryos and 6% of *itr-1(sa73) *embryos do not develop past the embryonic stage (Figure 7B). If ERL-1 negatively regulated ITR-1 activity, *erl-1(tm2703) *would be expected to decrease embryonic arrest in *itr-1(sa73) *mutants. However, *erl-1(tm2703) *did not significantly alter rates of embryonic arrest either in wild type animals or in *itr-1(sa73) *mutants (Figure 7B). Thus, despite its widespread presence in *C. elegans *embryos, ERL-1 is not essential for embryonic development and does not measurably affect ITR-1 signaling during this process.

#### (c) Defecation Rate

A particularly well studied function of ITR-1 is to control defecation rates. While ITR-1 LOF leads to increased defecation cycle lengths, ITR-1 GOF slightly decreases the length of the cycle [16,33]. ITR-1 functions in intestinal cells to control defecation rates [16] but since ERL-1 levels in the intestine are below detection limits (Figure 4C), it is unlikely that ERL-1 would affect this rhythmic behaviour by acting on ITR-1. Indeed, we did not observe any significant effect of *erl-1(tm2703) *on defecation rates in wild type, *unc-24(e138) *or *itr-1 *mutant strains (Figure 7B).

In summary, we investigated the effect of ERL-1 deficiency on three distinct IP3R-dependent processes but could find no evidence for a role of ERL-1 in negatively regulating IP3R activity.

### Lack of ERL-1 does not affect response to ER stress

**Figure 8:**
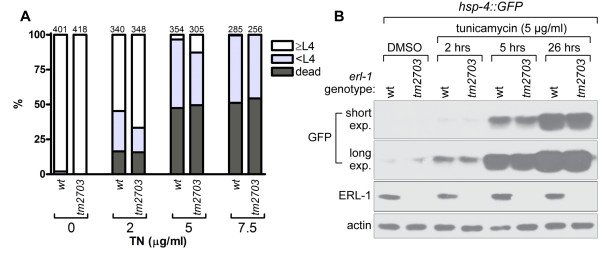
***erl-1(tm2703) *does not alter response to ER-stress**. (A) Wild type (wt) or *erl-1(tm2703) *embryos were plated onto NGM plates containing the indicated concentrations of tunicamycin (TN). After 72 hrs, animals were grouped into three categories (dead, < L4 and ≥ L4). Total number of animals scored are indicated above columns. (B) Mostly adult worms of *hsp-4::GFP *expressing strains (either *erl-1 *wt or *erl-1(tm2703)*) were plated onto NGM plates containing 5 μg/ml TN or DMSO only. Expression of *hsp-4::GFP *was determined by Western blotting using a GFP specific antibody. Western blots were also probed for ERL-1, demonstrating that ERL-1 protein levels are not altered by ER stress treatment (actin used as loading control).

In addition to targeting IP3Rs for ERAD, mammalian erlins have been shown to play a role in targeting certain other proteins for degradation by the ERAD pathway [3,5,7]. It is therefore possible that *C. elegans *ERL-1 might function in ERAD of a broad range of substrates beyond ITR-1 and indeed, ERL-1 might be involved in a more general clearance of unfolded proteins from the ER. *C. elegans *strains with mutations in ERAD show decreased survival and delayed development in the presence of ER stress inducing agents, like tunicamycin (TN) or dithiothreitol (DTT) [19,20,24,34]. To examine the effect of ERL-1 deficiency on TN sensitivity, we plated wild type and *erl-1(tm2703) *embryos onto NGM plates containing different concentrations of TN. After 72 hrs, worms were scored by dividing them into three categories: (1) dead, (2) younger than L4 (< L4) and (3) L4 and adults (≥L4). We detected no difference in development and survival between wild type and *erl-1(tm2703) *(Figure 8A). We performed a similar experiment with a second ER stressor, DTT, but again saw no significant difference. In more detail, wildtype and *erl-1(tm2703) *embryos were laid on standard NGM plates containing either 0 or 5 mM DTT. After 72+/-3 hours at 20°C, worms were collected and tip-to-tail lengths measured quantitatively (total of 427 worms scored). The three day exposure to 5 mM DTT reduced the average body length by a factor of 0.73+/- 0.26 and 0.74+/- 0.16 for wildtype worms and *erl-1(tm2703) *worms, respectively.

Mutations disrupting the ERAD pathway also increase levels of ER stress under basal and ER stress conditions [19,20,22-24]. ER stress levels can be monitored using a reporter construct, in which GFP expression is controlled by the promoter region of *hsp-4 *[18]. HSP-4 is the *C. elegans *homologue of the mammalian ER chaperone grp78/Bip and becomes transcriptionally upregulated in response to ER stress [18]. We examined GFP expression in *hsp-4::GFP *worms by Western blotting (Figure 8B). *erl-1(tm2703) *had no apparent effect on GFP expression either under basal conditions or following exposure to 5 μg/ml TN for various lengths of time. Many ERAD proteins become upregulated in response to ER stress [23,24,35] but ERL-1 protein levels were not affected by TN treatment (Figure 8B). In summary, our data indicate that ERL-1 does not play an essential role in the *C. elegans *ERAD pathway.

## Discussion

This study is the first to characterize the *C. elegans *erlin homologue and to explore erlin function in the context of an intact organism. We show that the *C. elegans *erlin homologue ERL-1 is highly similar to human erlins, both in sequence and in biochemical behaviour. Although such strong conservation across species suggests an important function for erlin proteins, lack of ERL-1 does not produce a detectable phenotype in *C. elegans*. Based on mammalian cell culture experiments, erlins have been implicated in ERAD of activated IP3 receptors [3,5] and thus might negatively regulate IP3R signalling. However, based on expression pattern alone, ERL-1 is unlikely to be a ubiquitous necessary regulator of ITR-1, the *C. elegans *homologue of IP3R.

We examined the effect of ERL-1 deficiency on three different ITR-1 dependent processes: embryonic development, brood size and defecation rates [15-17]. Since ERL-1 is widely expressed in the embryo, ERL-1 could potentially regulate ITR-1 activity during embryonic development. However, our data provide no evidence that ERL-1 regulates embryonic development, with or without involvement of ITR-1. IP3R signaling affects brood size by controlling contractions of the gonadal myoepithelial sheath cells as well as dilations of the spermatheca [31,32]. Immunofluorescence could not clearly establish if ERL-1 was expressed in the gonadal sheath cells or in the spermatheca. Thus, the lack of effect of ERL-1 on brood size could either be due to lack of expression in the appropriate tissue or simply because ERL-1 does not affect *itr-1 *activity during ovulation. Similarly, defecation rates are controlled by ITR-1 expressed in intestinal cells [16] but since ERL-1 is not expressed in the intestine, it was to be expected that we could detect no effect of ERL-1 loss on defecation rates. Overall, our results indicate that ERL-1 cannot be either a ubiquitous or a necessary regulator of ITR-1 dependent processes in *C. elegans*. Redundancy with similar proteins cannot explain this lack of effect because other SPFH proteins in *C. elegans *only share remote sequence similarity with erlins.

So, why does erlin loss in *C. elegans *have so few consequences compared to erlin loss in humans, which appears to cause serious disease [13]? Obviously, we cannot rule out subtle minor phenotypes in *C. elegans *nor can we rule out an unknown parallel pathway that could compensate for erlin loss. It is also possible that worms adapt to ERL-1 loss by upregulating other proteins. However, some of the different behaviour might reflect the different time scales on which worms and mammals operate their lives. In mammalian cells, proteasomal degradation of IP3Rs has only been observed after prolonged stimulation by ligands that induce a sustained increase in IP3 levels [36]. Degradation of IP3R protein in response to activation usually occurs over a period of several hours with a half maximal effect at 30-60 mins [37-40]. ERAD therefore appears to represent a negative regulatory feedback mechanism in processes involving sustained activation of IP3R. Such a global stimulation of IP3Rs by external application of artificially high concentrations of ligands cannot be achieved in *C. elegans*. In contrast, we investigated physiological processes involving IP3R activation. At least two of the processes investigated in the present study, gonadal sheath cell contractions and defecation cycles, involve cyclic IP3R activation on a much shorter timescale [16,41]. These processes require rapid activation and deactivation of IP3Rs, and deactivation has been shown to be at least partly mediated by enzymes that process IP3, such as IP3 kinase and IP3 phosphatase [15,32,42]. Thus, ERAD may not provide a sufficiently rapid mechanism for IP3R inactivation to play a role in processes such as *C. elegans *ovulation and defecation that occur on a time scale of minutes or even seconds.

## Conclusions

Erlins have been strongly implicated in ERAD-based turnover of IP3 receptors in mammalian cell cultures. We have searched for a similar function for the highly conserved erlin homolog (ERL-1) in the nematode *Caenorhabditis elegans*. Loss of function of the *C. elegans erl-1 *gene produces no obvious phenotype; in particular, we could find no evidence that ERL-1 participates in several IP3R based processes, such as ovulation, embryogenesis and defecation. Overall, we conclude that ERL-1 is unlikely to be a ubiquitous and necessary regulator of IP3R function in *C. elegans*.

## Methods

### Worm strains and handling

Breeding and maintenance of *C.elegans *stocks were performed according to standard procedures. The Bristol strain N2 was used as wild type strain [43]. Experiments were carried out at 20°C unless indicated otherwise. Strain FX2703 *erl-1(tm2703) *was obtained from the National Bioresource Project (Tokyo, Japan) and outcrossed five times before performing experiments. Strains JT73 *itr-1(sa73) *and SJ4005 *zcIs4[hsp-4::GFP] V *were obtained from the *Caenorhabditis *Genetics Center (University of Minnesota, Minneapolis, MN). Strains HR438 *unc-24(e138) *and HR762 *itr-1(sy290) unc-24(e138) *were kindly provided by Dr. Paul Mains (University of Calgary, Calgary, AB, Canada).

### Antibodies

We used the following commercially available antibodies: rat α-HA monoclonal antibody (3F10, Roche Applied Science), mouse α-actin clone C4 (MAB1501, Millipore), rabbit α-calnexin (SPA-860, Stressgen), horseradish peroxidase conjugated goat α-rabbit, goat α-mouse and goat α-rat IgGs (Santa Cruz Biotechnology), goat α-rat IgG AlexaFluor 488, goat α-mouse IgG AlexaFluor 488 and donkey α-rabbit IgG AlexaFluor 568 (Molecular Probes). Rabbit α-GFP antibody was kindly provided by Luc Berthiaume (University of Alberta, Edmonton, Canada)

The polyclonal antibody against ERL-1 was raised by immunizing rabbits with His-tagged ERL-1(182-312) and affinity purified using a glutathione S-transferase tagged version of the same antigen cross-linked to Glutathione Sepharose 4B (GE Healthcare) [44].

### Plasmid constructs and transfection of cell lines

ERL-1HA (wild type) and ERL-1(182-312) were cloned by PCR using as template the *erl-1 *cDNA clone yk705a8 (kindly provided by Yuji Kohara, National Institute of Genetics, Mishima, Japan) as a template. HA-tagged constructs were cloned into pLPCX (Clontech) using XhoI and ClaI restriction sites. ERL-1 F303A HA was generated from wild type ERL-1HA/pLPCX by DpnI-mediated site-directed mutagenesis. His- and GST-tagged versions of ERL-1(182-312) were generated by cloning the PCR product into pTrcHis C (Invitrogen) using BamHI and PstI restriction sites or into pGEX-2T (GE Healthcare) using BamHI and SmaI sites respectively. HeLa and HEK293 cells were maintained in Dulbecco's Modified Eagle's Medium supplemented with 10% fetal bovine serum. Transient transfection of cell lines with ERL-HA constructs was performed using Fugene6 (Roche Applied Science) according to the manufacturer's instructions.

Transcriptional reporter constructs were generated by cloning putative *erl-1 *promoter regions (relative to *erl-1 *start codon: -182 to +1; -1022 to +1; -1022 to +576) 5' of a nuclear-targeted GFP reporter plasmid (pJM355). Plasmids were injected at a concentration of 100 μg/ml (together with the *unc-119 *rescuing plasmid pDP#MM016B at the same concentration) into the syncytial gonads of *unc-119(ed4) *hermaphrodites. Transformed worms were identified and strains propagated on the basis of*unc-119 *rescue.

### Reverse transcriptase (RT)-PCR

Total RNA was isolated from mixed stage worms using TRIZOL reagent (Invitrogen). Reverse transcription was performed using the SuperScript RT-PCR system (Invitrogen) with oligo(dT) primers. *erl-1 *cDNA was amplified by PCR (forward primer: ATGCTAACCGAGTTGGCGCT; reverse primer: GGATGAGGCGTGACAGGTAT), cloned into pGEM-T easy (Promega) and sequenced. Amplification of the *erl-1 *coding region from wild type cDNA yielded the expected product of 1000 bp (Figure 3C). However, PCR of *erl-1(tm2703) *cDNA with primers designed to amplify the *erl-1 *coding region from the transcription start site to the 3'UTR resulted in a product of ~700 bp. This was slightly larger than the predicted size of the mutant spliced mRNA but sequencing showed that the spliced *erl-1(tm2703) *mRNA also contained part of the first intron. This explained the difference between predicted and observed size of *erl-1(tm2703) cDNA *and is likely due to loss of a splice acceptor site in the mutant transcript.

### Immunofluorescence staining

Immunofluorescence staining of cell lines and dissected gonads and intestines was performed as described previously [4,45]. Hypochlorite treated embryos were permeabilized using the freeze crack method [46]. Slides were fixed with ice-cold methanol and acetone (5 mins each) and rehydrated in a series of alcohols. Phosphate buffered saline (PBS) with 5% bovine serum albumin (Roche) and 0.1% Triton X-100 (Sigma) was used for blocking and antibody dilution. Incubation with primary antibodies was performed overnight at 4°C. Slides were stained with affinity purified α-ERL-1 and mouse α-actin. The latter antibody was used as a control for antibody penetration. Slides were incubated with secondary antibody for 1 hour at room temperature. After each antibody incubation, slides were washed three times for 10 minutes in 0.1% Triton X-100 in PBS. Slides were mounted using fluorescent mounting media (Dako). Confocal images of HeLa cells and *C. elegans *embryos were acquired as Z-stacks using an LSM 510 Meta confocal on an Axiovert 200 M microscope with a 63×/1.4 Plan Apochromat objective (all Zeiss). Confocal images are presented as projections of three focal planes generated using LSM images browser (Carl Zeiss). Images of dissected gonads and intestines were acquired on Zeiss Imager Z1 microscope equipped with an Axiocam MRM digital camera using an EC Plan-Neofluar 40×/1.30 Oil DIC M27 objective. Non-specific background staining was determined by parallel staining of *erl-1(tm2703) *samples. For presentation purposes, levels, contrast and brightness were adjusted across the entire image using Adobe Photoshop. Identical settings were used for acquisition and processing of images of wild type and *erl-1(tm2703) *samples.

### Preparation of protein samples and Western blotting

Sucrose gradient centrifugation was performed according to a previously published protocol [4]. For preparation of *C. elegans *protein samples, worms were harvested and washed in ddH20 and frozen at -80°C. Frozen pellets were resuspended in lysis buffer (1% Triton X-100, 1% sodium deoxycholate, 0.1% SDS, 10% glycerol, 150 mM NaCl, 10 mM Tris-HCl, pH 8.0) containing protease inhibitors (1 mM phenylmethylsulfonyl fluoride, 10 μg/ml each of aprotinin and leupeptin) and homogenized by sonication. Lysates were cleared by centrifugation at 16,000 × g for 10 mins at 4°C. Equal amounts of protein were loaded onto sodium dodecyl sulfate polyacrylamide gel electrophoresis (SDS-PAGE) gels and Western blot analysis was performed using standard procedures.

### Phenotypic characterization

For measuring growth rate, gravid one day old adults were allowed to lay eggs on NGM plates for two hours. Adults were subsequently removed and plates were kept at 20 or 26°C. Images of developing larvae were acquired every 24 hrs for four days using a Canon PC1210 digital camera mounted onto a Zeiss Stemi SV11 dissecting microscope. Lengths of worms were measured using ImageJ version 1.42 q (National Institutes of Health, USA). Data presented here show results from one experiment, but experiment was repeated once with almost identical results.

To determine life span, L4 animals were picked and transferred onto a fresh plate every 2 days. Animals were considered dead when no movement in response to touch was observed. Between 21 and 27 animals in two independent experiments were scored per strain.

Brood size was determined by picking L4 animals (two animals per plate) and transferring these to a fresh plate every 24 hours until egg laying ceased. Offspring were counted two days after mothers were removed from plates. Individual brood size was calculated from the average brood size of two mothers on each plate. Rates of embryonic arrest were determined by counting unhatched embryos 24 hrs after removal of mothers.

Defecation rates of first day adults grown at 20°C were determined by measuring times between posterior body contractions. During measurements, plates were placed on top of a petri dish containing cold water to serve as a heat sink. For each strain, we measured on average six defecation cycles for each of five worms. Brood size, embryonic arrest and defecation data were collected in two rounds of experiments.

Results depicted as bar graphs represent means +/-SD. For multiple comparisons a one-way ANOVA with Newman-Keuls post test was applied.

### ER stress experiments

To assess sensitivity of worms to tunicamycin (TN, Calbiochem), first day gravid adults were allowed to lay eggs for ~4 hours on plates containing different concentrations of TN. After 72 hours, plates were scored by dividing worms into three categories: (1) dead, (2) < L4 and (3) ≥ L4. Combined results from three independent experiments are shown here. Levels of ER stress were determined by plating mixed stage *zcIs4[hsp-4::GFP] *worms onto plates containing 5 μg/ml TN for the times indicated. GFP expression was analyzed by Western blotting. Experiment was performed twice.

## Authors' contributions

MBH conceived the study, designed and performed experiments, analyzed data and drafted the manuscript. JDM conceived the study, designed experiments, carried out worm injections, analyzed data and helped draft the manuscript. SMR conceived the study. All authors read and approved the final manuscript.

## Supplementary Material

Additional File 1 **Figure S1. *erl-1 *reporter constructs**. Localization of genomic sequences used for reporter constructs (yellow boxes) within the *erl-1 *containing operon. Schematic of operon was downloaded from Wormbase version 221 http://www.wormbase.org. Click here for file
