# Supplementary figures and images for: Characterization of the C. elegans erlin homologue
Source: BMC Cell Biol. 2012 Jan 23;13:2. doi: 10.1186/1471-2121-13-2 (PMC3292932; doi:10.1186/1471-2121-13-2)

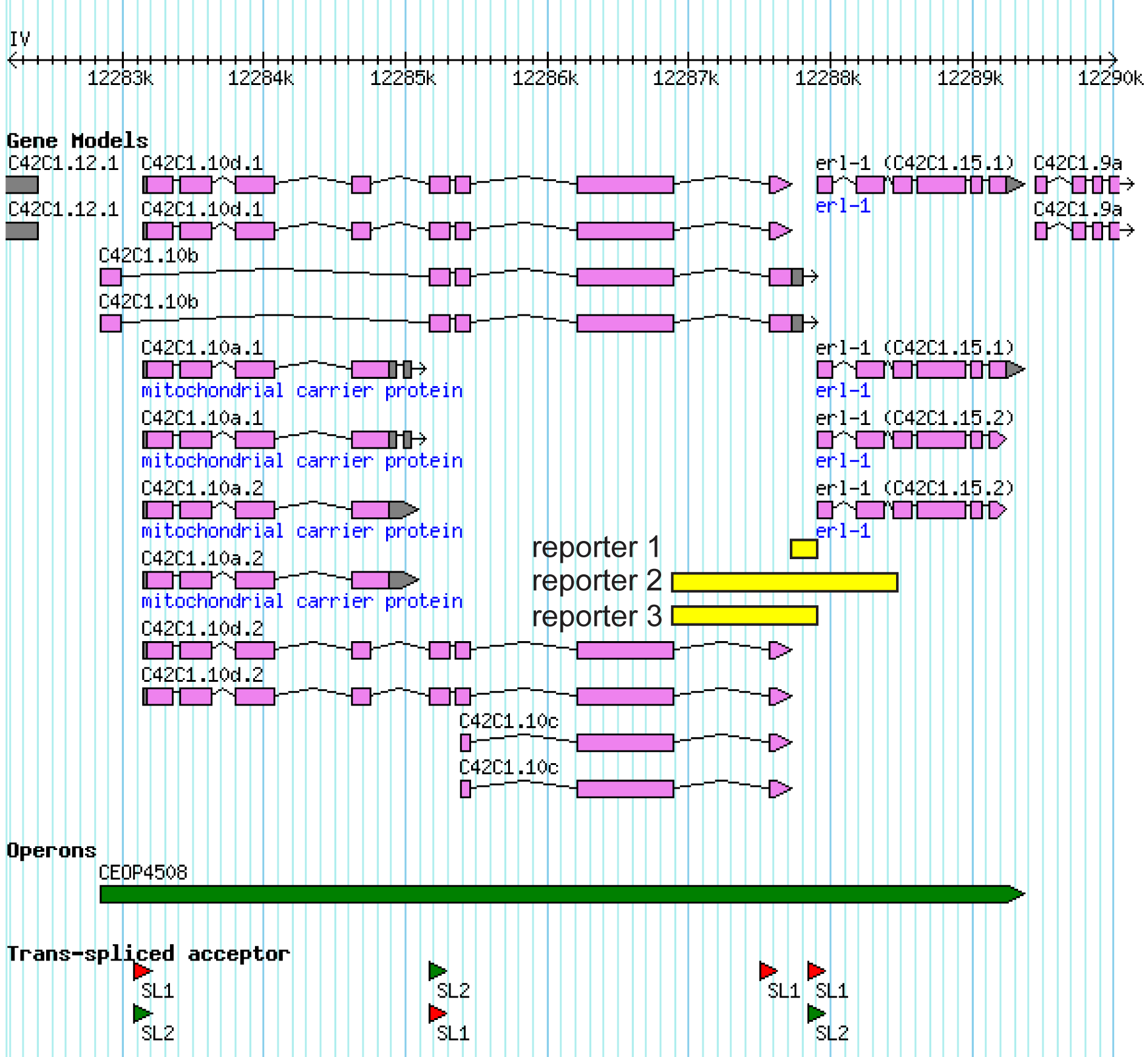

Supplement: Additional File 1 — Figure S1. erl-1 reporter constructs. Localization of genomic sequences used for reporter constructs (yellow boxes) within the erl-1 containing operon. Schematic of operon was downloaded from Wormbase version 221 http://www.wormbase.org. [file 1471-2121-13-2-S1.TIFF]
